# Drug Screening of Sarcoma Cells: Finding Shared Sensitivities

**DOI:** 10.1158/2767-9764.CRC-26-0142

**Published:** 2026-06-17

**Authors:** Hannah C. Beird, Carl Ho, Roberto Cardenas-Zuniga, Asmaa Ahmed, Clement Agyemang, Danh D. Truong, Kevin Murgas, Clifford C. Stephan, Yong Sung Park, Reid T. Powell, Joseph Tym, Sharon M. Landers, Stephanie Schmidt, Noha M. Osman, Dejka M. Araujo, Anthony P. Conley, Neeta Somaiah, Bissan Al-Lazikani, Joseph A. Ludwig, Andrew Futreal

**Affiliations:** 1 Department of Genomic Medicine, The University of Texas MD Anderson Cancer Center, Houston, Texas.; 2 Sarcoma Medical Oncology, The University of Texas MD Anderson Cancer Center, Houston, Texas.; 3 High-Throughput Research and Screening Center, Center for Translational Cancer Research, Texas A&M Health Science Center, Institute of Biosciences and Technology, Houston, Texas.; 4 The Institute of Cancer Research, London, United Kingdom.; 5 Surgical Medical Oncology, The University of Texas MD Anderson Cancer Center, Houston, Texas.

## Abstract

Many of the >130 World Health Organization–defined sarcoma subtypes are chemoresistant. To identify drug candidates with potential for repurposing in sarcomas with unmet needs, we screened structurally diverse small molecules across 20 sarcoma cell lines of various histologies. The screen included 1,387 compounds from the Custom Clinical Collection, NCI-Approved Oncology Set IV, and Selleck Bioactive Collection. The most effective agents were microtubule inhibitors, aurora kinase inhibitors, and heat shock protein inhibitors. Differential drug sensitivity was observed by sarcoma subtype. Ewing sarcoma, for example, was sensitive to aurora kinases and to TORIN-2 (an mTOR inhibitor). In contrast, myxoid liposarcoma and synovial sarcomas exhibited marked sensitivity to SNS-032, a CDK2/7/9 inhibitor identified by our screen. Interestingly, SNS-032 abrogates the downstream YAP pathway, a known driver pathway in the growth of these histotypes. Therefore, similar drug responses across histologies may indicate shared driver pathways and vulnerabilities.

**Significance:**

Sarcoma cells are resistant to most compounds that are currently approved for treating various diseases. Ewing sarcoma cells have distinct sensitivities to aurora kinases that can be explored, whereas myxoid liposarcoma and synovial sarcoma cells share similar drug sensitivity fingerprints that could be exploited in future basket trials.

## Introduction

Although sarcomas represent <1% of all cancers, their prevalence among adolescents and young adults imposes a significant cancer burden on society. More than 130 sarcoma subtypes exist, with molecular and clinical distinctions that can be broadly categorized by fusion protein status, clinical aggressiveness, and chemosensitivity (1, 2). Some success has been achieved in devising treatment strategies based on actionable point mutations (3). However, the point mutation rate is generally low in these tumors, and few patients benefit from this triage (4). Due to their rarity, soft-tissue sarcoma–specific clinical trials can be challenging to complete because of slow accrual. As an alternative to subtype-specific trials, ongoing histotype-agnostic basket trials target shared genetic aberrations (e.g., loss of *TP53* and *RB1* and mutations in *ATRX*).

Other efforts are being developed to repurpose approved therapies for sarcomas. In this approach, chemical screening of existing sarcoma models is performed to identify subtype-agnostic drug vulnerabilities. Three recent large-scale drug screens have postulated these possible candidates. For instance, a screen testing 445 agents on 63 sarcoma cell lines found that both Ewing sarcoma and synovial sarcoma cells are sensitive to aurora kinases and that several histologies were sensitive to MEK kinase inhibitors (1). A second screen of 525 anticancer agents across 15 sarcoma cell lines suggested that patients with sarcoma with translocations could be treated with the kinase inhibitor dasatinib, which inhibits BCR-ABL, SRC family, c-KIT, EPHA2, and PDGFRB (2). The most recent screening of 194 patient-derived tumor organoids from 126 patients spanning 24 sarcoma subtypes revealed that several Food and Drug Administration (FDA)–approved compounds not currently used in sarcoma standard of care may be effective (3). Here, we conducted an *in vitro* drug screen with 1,387 drugs across 20 sarcoma cell lines from various histologies to determine subtype-agnostic drug vulnerabilities. We have included myxoid liposarcoma, which has not been previously tested at this magnitude. The pan-sarcoma analysis served to gain insights into shared sensitivities and resistances across histologies.

## Materials and Methods

### Cells

For the screen, we employed the following cells: chondrosarcoma (CS) SW1353 (CVCL_0543), CAL78 (CVCL_1809), and HEMCSS (CVCL_1238); fibrosarcoma (FS) HT1080 (CVCL_0317); SS SW982 (CVCL_1734), SYO-1 (CVCL_7146), and HS-SYII (CVCL_8719); Ewing sarcoma A4573 (CVCL_6245), EW8 (CVCL_1204), TC32 (CVCL_7151), and TC71 (CVCL_2213); dedifferentiated liposarcoma (DDLPS) Lipo224A, Lipo573B, Lipo863B, and Lipo246; pleomorphic liposarcoma (PLS) PLS-1 (PLS-DL-812); myxoid liposarcoma MLS-1765-92 (CVCL_S817), MLS-402-91 (CVCL_S813), and DL-221 (CVCL_DQ91); and osteosarcoma LM7 (CVCL_0515). Ewing sarcoma cells A4573 and EW8 were purchased from the American Type Culture Collection and were obtained from the Cytogenetics and Cell Authentication Core Facility at the University of Texas (UT) MD Anderson Cancer Center. DDLPS lines PLS-1, DL-221, and LM7 were developed at the UT MD Anderson Cancer Center (4–6). Myxoid liposarcoma cells MLS-1765-92 and MLS-402-91 were a gift from Pierre Aman. It is to be noted that HT-1080 is a FS of the bone that was derived from a dedifferentiated CS (7, 8). Cells were cultured in DMEM supplemented with 10% fetal bovine serum (FBS; Gemini Bio-Products) and 1% penicillin–streptomycin (100 U/mL) and placed in a humidified 5% CO_2_ incubator at 37°C. MLS cell lines 402-91 and 1765-92 were cultured in RPMI 1640 (Thermo Fisher Scientific) supplemented with FBS and antibiotics. MLS-1765-92 *FUS::DDIT3* has translocation type 8 fusion (730 bp), whereas the MLS-402-91 *FUS::DDIT3* has translocation type 1 (136 bp). DL-221 has a *FUS::DDIT3* translocation of type 1 (136 bp). All cell lines were tested for mycoplasma infection every 6 months, and short tandem repeat (STR) DNA fingerprinting was performed on the cell lines.

### Drug libraries

The drug panels were sourced from (i) Custom Clinical Collection of agents currently used in the clinic to treat various forms of cancer and clinical trials, (ii) NCI Approved Oncology Set IV of FDA-approved anticancer drugs, and (iii) Selleck Bioactive Collection of FDA-approved inhibitors, active pharmaceutical ingredients, natural products, and chemotherapeutic agents. These panels are structurally diverse, medicinally active, and cell-permeable. Compounds were diluted to 10 mmol/L in DMSO.

### Drug screen growth assay

Growth curves were performed for all cell lines. Cells were seeded in twofold dilutions from 2,000 to 125 cells/well in 50 μL of growth media specific for each cell line. Cell counts were performed daily. The DNA in the nucleus of cells in six to eight technical replicate wells from each plating density was stained with 5 μg/mL Hoechst 33342 for 10 minutes at 37°C. A single 4× fluorescent image [Nikon Plan Apo 4x/0.20 NA dry lens, 405 nm ex/455 (55) nm em] was collected from each well with a GE IN Cell 6000 Analyzer (GE Healthcare Life Sciences) automated confocal microscope. Nuclei in each image were segmented based on size and intensity and counted. The cellular growth rate was calculated, and an appropriate plating density was selected so that the cells were in log-phase growth and the number of cells in the well on the final day of the screening assay would be 70% to 80% confluence or less.

### High-throughput screening assay

Cells were plated at a density (400–3,000 cells per well) determined by a growth assay such that they would be 70% to 80% confluent within 72 hours after compound treatment. Cells were plated using a Multidrop Combi dispenser (Thermo Fisher Scientific). Cells were plated in 50 μL of growth media per well into black 384-well tissue culture-treated plates with flat, clear optical plastic bottoms (Greiner Bio-One, cat. #781091). Cells were left at room temperature for 40 to 60 minutes before being placed into humidified cell culture incubators. Humidity within the incubators was kept at or above 95% relative humidity, as determined with a Monarch Instruments temperature and humidity Track-It data logger (VWR International; cat. #89184-574), to minimize the possibility of edge effects during the assay. Cells were kept overnight at 37°C and 5% CO_2_ before the addition of 50 nL of compound by pin transfer from 1,000× DMSO stocks using a Beckman Coulter Biomek FX (Beckman Coulter) equipped with a 50 nL pintool from V&P Scientific. Plates were processed for imaging by washing the wells twice with 100 μL/well phosphate-buffered saline (PBS, Teknova Inc.; cat. #P3195) at room temperature using a HydroSpeed plate washer (Tecan), followed by the addition of a mixture of 1% paraformaldehyde and 0.1% glutaraldehyde (Electron Microscopy Sciences) using a Multidrop Combi for 30 minutes at room temperature. Cells were washed once again with 100 μL/well PBS, and the nuclei were stained for 30 minutes with 1 μg/mL 4′, 6-diamidino-2-phenylindole (DAPI, Thermo Fisher Scientific; cat. #D1306). Cells were washed a final time with 100 μL of PBS per well and left in PBS for imaging. Brightfield and fluorescent DAPI images (4×) were sequentially collected from each well using the IN Cell 6000 Analyzer. DAPI-stained nuclei were segmented and counted using the prebuilt Cell Count algorithm in the IN Cell Developer version 1.6 (GE Healthcare Life Sciences). Cell nuclei >25 μm^2^ were the primary criterion, followed by debris filtering to remove noncellular objects based on morphology and intensity and cell counting. Before batch analysis of each sarcoma cell line, we always reviewed the existing segmentation parameters to ensure compatibility with the morphology of the new cell line. When required, settings such as diameter and intensity cutoff values were adjusted to optimize detection. Therefore, although a consistent initial analysis method was employed, final parameter settings were individually confirmed for each cell line and modified as needed. In most instances, duplicate biological replicate screens were run on consecutive weeks.

Staurosporine or etoposide was run as an internal positive control on all screening plates and included as an eight-point concentration response curve in duplicate on each assay plate. A z-prime statistic (9) was calculated for each assay plate using the on-plate drug-treated positive and DMSO-treated negative control wells. A minimum significant ratio (10) was calculated for the eight-point control concentration–response curves and compared across all the assay plates in the screen. A comparison of the well-to-well standard deviations over the two replicate screens was also performed to evaluate the screens for high variability between runs. These were calculated but not tabulated; they were only used to display error heat maps. Mean squared error was used to determine confidence on a compound-by-compound basis and incorporated into classification criteria, as outlined below. The numerical results from image analysis of the microtiter plates are first normalized to intraplate controls. Any plates that fail validation are repeated. Dose–response characteristics are determined by fitting a four-parameter logistic (“Hill”) equation to the fraction affected metric of compound response values, and the area under the curve (AUC) is computed using numerical integration. AUC values are then normalized to a range of 0 to 1 by dividing the AUC values by the number of logs over which the compounds were tested (5 logs, from 10^−10^ to 10^−5^).

### Global analyses using AUCs

AUC values were filtered to identify the most selective compounds. First, we excluded drugs that showed broad or widespread inhibition, which would likely indicate generalized cytotoxic mechanisms of action and elicit toxicity in patients. For this, a cutoff was set for the maximum mean AUC of 0.7. Second, drugs with little activity across all cell lines were also excluded using a minimum mean AUC cutoff of 0.1 (Supplementary Table S1). The AUCs were ranked for each cell line. The top-ranking AUCs (top 30th or 90th percentiles) were used to summarize drug classes across all cells and by the major histologies represented. UpSet plot data were extracted from the UpSetR package (11). Unsupervised hierarchical clustering was performed with the pheatmap package in R (12).

### Validation growth assay

Myxoid liposarcoma has not been tested in large-scale drug screens. Thus, MLS-1765-92 (CVCL_S817), MLS-402-91 (CVCL_S813), and DL-221 (CVCL_DQ91) were selected to assess the effects of SNS-032 on cell proliferation and Western blotting. MLS-1765-92 and MLS-402-91 cell lines were cultured in RPMI 1640 supplemented with 10% FBS and 1% penicillin–streptomycin. DL-221 was cultured in DMEM supplemented with 10% FBS and 1% penicillin–streptomycin. All cell lines were tested for mycoplasma contamination and authenticated by STR DNA fingerprinting before use in cell culture.

### *In vitro* analysis of SNS-032

From 80% to 90% confluent T-150 cm^2^ flasks, each cell line was detached with 0.25% trypsin at 37°C for 5 minutes. Then, cells were suspended in RPMI 1640 media supplemented with 10% FBS and 1% penicillin–streptomycin and placed in conical tubes. Cells were pelleted at 3,000 rpm for 5 minutes, and media was replaced with fresh, complete corresponding media. Cells were counted to plate 2 × 10^3^ cells in a 96-well plate in complete media. Cells were incubated at 37°C overnight. The next day, the corresponding concentration of SNS-032 was added to the wells. In brief, ranging from 20 nmol/L to 10 μmol/L of SNS-032, 10 serial twofold dilutions were added to the corresponding wells in the plates (0.5% DMSO final volume). Cells were incubated at 37°C for 72 hours. After that, the viability was measured using Alamar Blue reagent. Then, cells were incubated at 37°C for at least 4 hours, and absorbance was measured at 570 nm. Data were analyzed using GraphPad Prism version 10 software, IC_50_ values were calculated, and averages of three independent replicates were obtained.

### Western blotting

After treating myxoid liposarcoma cell lines (MLS-1765-92, MLS-402-91, and DL-221) with 200 nmol/L SNS-032 and DMSO for 6 hours, the cell lysates were isolated. After protein concentration normalization, samples (2 mg/mL protein) were prepared for analysis using the Jess Simple Western system (ProteinSimple). The prepared samples were loaded, separated, immobilized, and incubated with primary antibodies against YAP1 (cat. #D1224, 1:50), TAZ (cat. #ab110239, 1:10), CTGF (cat. #C2724, 1:50), and CYR61 (cat. #14479S, 1:50). The system was then incubated with horseradish peroxidase–conjugated secondary anti-rabbit IgG or anti-mouse IgG (cat. #DM-002, 1:100). Chemiluminescent detection was performed using the Simple Western detection module on JESS. The corrected AUC of each sample from the three replicates was extracted using Compass software (ProteinSimple).

Data were then statistically analyzed to determine the significance of differences in expression levels of each protein between DMSO control and SNS-032–treated sarcoma cell lines (*n* = 3 independent biological replicates). Raw AUC values were log_2_-transformed prior to statistical testing to account for variance proportional to the mean. For each of the four proteins, an independent two-way ANOVA was applied to evaluate the main effects of treatment, cell line, and their interaction. Pairwise comparisons of treatment within each cell line were performed to extract simple main effects, including percentage change and *post hoc* significance. To control the familywise error rate across the three cell lines, Bonferroni correction was applied to the *post hoc* tests. Statistical significance was defined as *P* < 0.05. All analyses were performed using R statistical software version 4.5.1, using the base *aov* function for ANOVA and the emmeans package (version 2.0.2) *emmeans* function for *post hoc* comparisons.

### RNA analysis

RNA sequencing data for cancer cell lines were obtained through the Cancer Cell Line Encyclopedia sequencing data made available through the DepMap portal, CTRPv2.0 (13). Data for sarcoma cell lines relevant to this article were extracted and analyzed as is. The gene expression values for *CDK2*, *CDK7*, and *CDK9* were visualized using the R package ComplexHeatmap (14).

## Results

**Figure 1.**
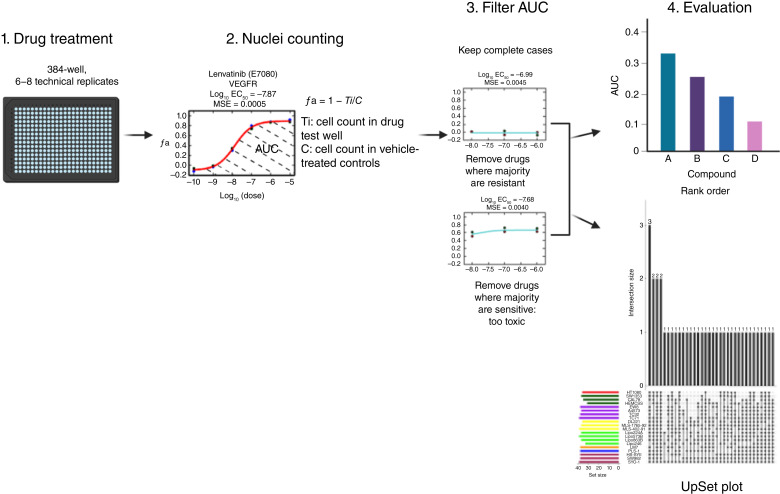
Schema depicting the experimental flow and analysis of the drug screen. [Created in BioRender. H.C. Beird (2026) https://app.biorender.com/illustrations/68966f6a318625625adb1570?slideId=b6742edb-4b0a-44f7-9f6b-8637d1616983.]

Here, we tested collections of both FDA-approved and clinical trial drug candidates (*N* = 1,387 drugs) for their inhibitory effects against 20 different human sarcoma cell lines: CS, Ewing sarcoma, DDLPS, FS, myxoid liposarcoma, PLS, SS, and osteosarcoma. Serially diluted cells were treated with each compound, and their proliferation was measured by counting nuclei (see “Materials and Methods” and Fig. 1). The dose–response curves were taken, and the AUCs were calculated, ranging from 0 to 1. An AUC of zero indicated complete ineffectiveness, whereas an AUC of one indicated potent inhibition of proliferation, interpreted as high drug sensitivity.

Next, filtering of the AUC values was performed to identify the most selective compounds. We excluded drugs that showed broad or widespread inhibition, indicative of generalized, cytotoxic mechanisms of action likely to harm tumor and normal cells alike and therefore induce significant toxicity in patients. For this, we set a cutoff with a maximum mean AUC of 0.7. We also excluded drugs that exhibited minimal activity across all cell lines using a minimum mean AUC cutoff of 0.1 (Supplementary Table S1). This yielded 217 compounds with AUC values between 0.1 and 0.7.

**Figure 2.**
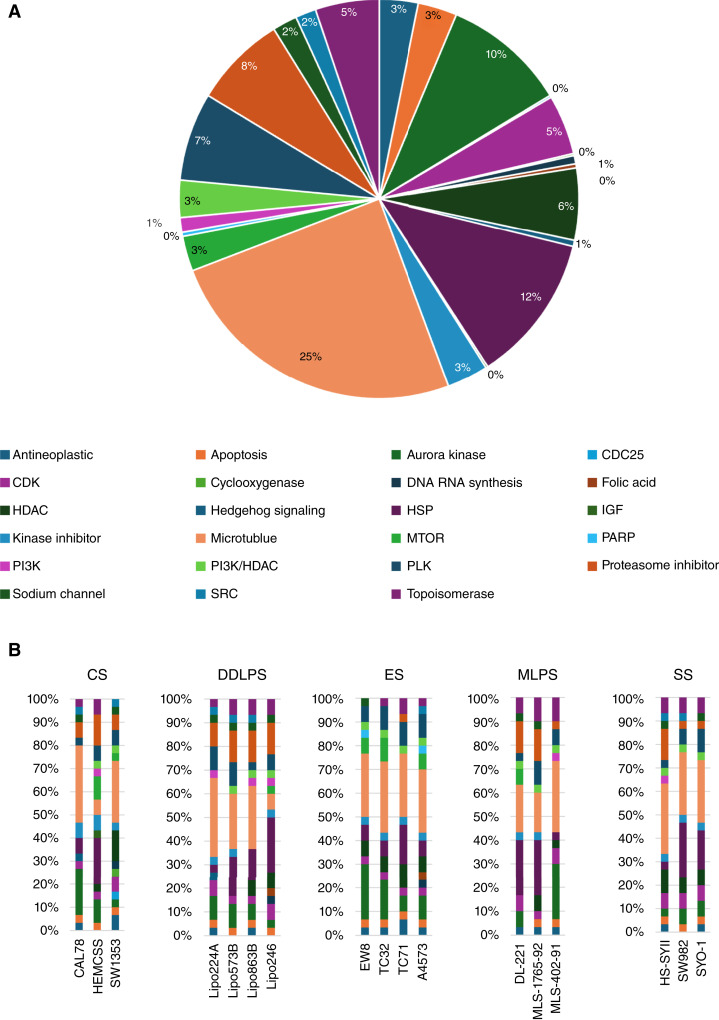
Summary of drug classes to which sarcoma cells were most sensitive. **A,** Pie chart displaying the proportions of drugs belonging to each class listed. **B,** Stacked bar plot for each major histologic cell line according to drug class. ES, Ewing sarcoma; MLPS, myxoid liposarcoma; SS, synovial sarcoma.

**Figure 3.**
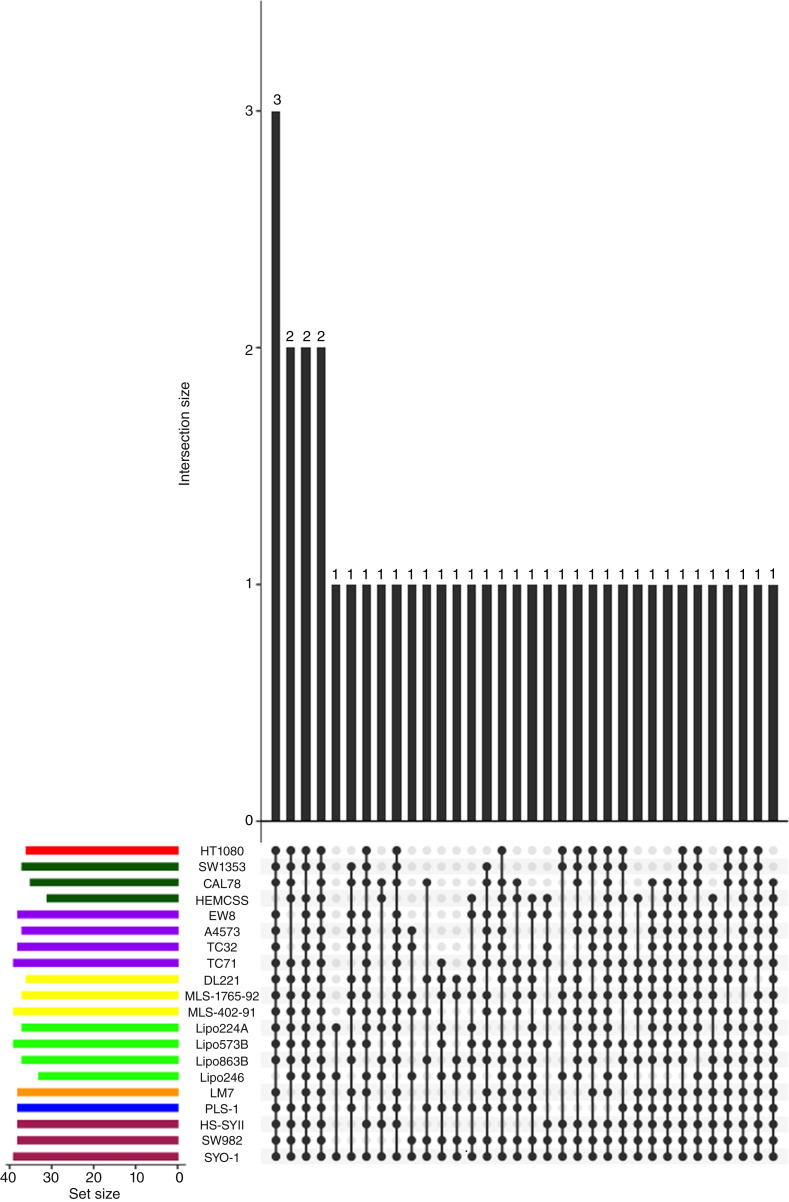
Overlapping drug sensitivities within the 90th percentile in drug sensitivity for each cell line. An UpSet plot is shown with annotation of the drug class (first column) and cell lines color-coded by histology.

We ranked this subset of agents by their AUC values for each cell line, using the sum of ranks (Supplementary Table S2). In this list, the most frequent class of drugs sensitive across multiple cell lines targeted microtubules, followed by drugs targeting heat shock proteins and aurora kinases (Fig. 2A). The top-ranking drug was a CDK inhibitor, dinaciclib. When examining these top-ranked drugs according to histologies with >1 cell line, we observed that sensitivity to aurora kinases was most prevalent in Ewing sarcoma (Fig. 2B). Next, all drugs within the 90th percentile in drug sensitivity for each cell line were taken and used in an upset plot to identify overlapping sensitivities across histologies (Fig. 3; Supplementary Table S3). Again, the most sensitive drug across all 20 lines was the CDK1/2/5/9 inhibitor dinaciclib, followed by the PI3K inhibitor CUDC-907. The MTOR inhibitor TORIN-2 was especially sensitive in Ewing sarcoma cells.

In transcriptomic analyses, gene expression profiles become enriched when cellular pathways are strongly activated. These gene signatures have been used to classify tumors into subgroups, often reflecting specific gene mutations or cellular states. Ultimately, they reflect tumor-promoting biological pathways that are key drivers within the tumors. We hypothesized that sensitivity would increase for drug targets that are members of key tumor-promoting pathways. These would not be broadly cytotoxic across all cells, promoting unwanted side effects in patients, but rather compounds selective for tumor-specific features. Conversely, resistance to multiple drugs with similar drug target classes may reveal inherent redundancies and cross-talk among pathways. One example is the MAPK pathway, which intersects with many growth factor pathways, such that inhibition of one growth factor would be less effective if it were concomitantly inhibited by another related growth factor. High and low drug-sensitivity measures could be combined into a drug-sensitivity fingerprint. We could expect that similar fingerprints are shared across various sarcoma histologies due to common driver pathways. If identified, these shared pathways could indicate the basis for combining histologies into basket trials. One advantage of identifying these shared sensitivity pathways through mining drug response data rather than gene expression data is that tissue specificity is ruled out. That is, tissue-specific markers in gene expression data often delineate many sarcoma subtypes from others, especially leiomyosarcoma (muscle-specific markers) and gastrointestinal stromal tumors (15). These markers may not reveal insights into the actual therapeutic vulnerabilities and often create extraneous signals. Thus, drug response data can mitigate the need to sift through these features to identify candidate driver targets.

We generated a heatmap based on AUCs (Supplementary Fig. S1) to investigate drug fingerprints. Ewing sarcoma lines clustered tightly together, having very similar sensitivity profiles. Myxoid liposarcoma and synovial sarcoma also clustered closely together. One of the drugs displaying shared sensitivity across every myxoid liposarcoma and synovial sarcoma line tested was SNS-032. SNS-032 is an inhibitor of CDK2/7/9, which leads to the dephosphorylation of serines 2 and 7 in the C-terminal domain of RNA polymerase II (RNAPolII), preventing transcriptional activity (16, 17). In many cancer types, including chronic lymphocytic leukemia (CLL), acute myeloid leukemia, high-grade serous ovarian cancer, esophageal carcinoma, and uveal melanoma cells, SNS-032 induces apoptosis and cell-cycle arrest (16–19). The SNS-032 targets CDK2/7/9 are expressed in synovial sarcoma lines Fuji and SYO-1 (20) and in sarcoma lines within the CCLE (Supplementary Fig. S2). For Fuji and SYO-1, inhibition of CDK7 by BS-181 resulted in decreased phosphorylation of RNAPolII at Ser5, with no large changes in RNAP II Ser2 phosphorylation (20).

CDK inhibition in sarcomas is being tested in the clinic (e.g., NCT04040205, NCT05538572, NCT05159518). Given that the top-ranked sensitive drug was the CDK inhibitor dinaciclib and the interesting, shared sensitivities of myxoid liposarcoma and synovial sarcoma to the CDK inhibitor SNS-032, we investigated one downstream effect of SNS-032. In uveal melanoma, SNS-032 inhibits the YAP pathway (21), which is also a key pathway downstream of the driver translocations in both myxoid liposarcoma and synovial sarcoma (22, 23). Nuclear staining of YAP1 is highest in these histologies compared with other sarcomas (24), and inhibiting *YAP1* expression in both synovial sarcoma and myxoid liposarcoma cell lines decreases proliferation (22, 23). Therefore, we hypothesized that YAP activity is abrogated in these histologies when treated with SNS-032. We first confirmed the drug sensitivity of myxoid liposarcoma to SNS-032 using an *in vitro* dose–response assay. The averages of three independent experiments in the myxoid liposarcoma cell lines (Fig. 2) showed that the IC_50_ values were 198 nmol/L (±23) for DL-221, 235 nmol/L (±17) for MLS-402-91, and, lastly, 351 nmol/L (±1) for MLS-1765-92.

**Figure 4.**
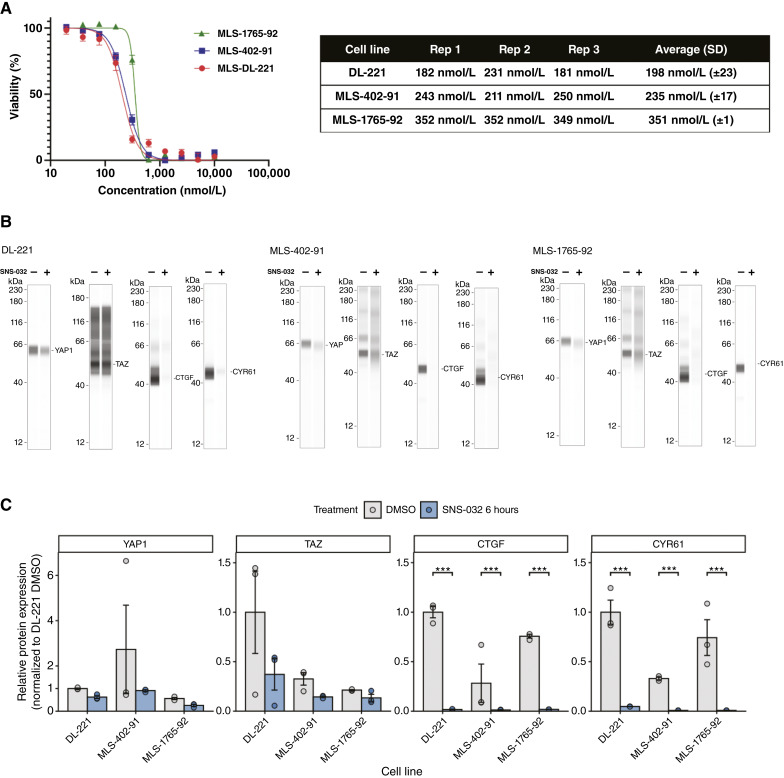
SNS-032 characterization in myxoid liposarcoma. **A,***In vitro* sensitivity of myxoid liposarcoma cell lines to the CDK inhibitor SNS-032. Myxoid liposarcoma cell lines were exposed to serial dilutions (1:2 factor) of SNS-032 ranging from 20 nmol/L to 10 μmol/L for 72 hours. Viability was measured using Alamar Blue reagent. Data were analyzed using GraphPad Prism version 10 software; IC_50_ values were calculated, and averages of three independent replicates were obtained. Summary of each IC_50_ and averages for all myxoid liposarcoma is provided. **B,** Treatment of myxoid liposarcoma cell lines with SNS-032 results in decreased YAP pathway activity. Cell lines were treated with 200 nmol/L SNS-032 for 6 hours, after which Western blotting was performed to quantify the protein levels of YAP1, its transcription partner TAZ, and two downstream effectors, CTGF and CYR61. Loading controls are shown in Supplementary Fig. S3. **C,** Quantification of protein levels from **B**. Bar graphs display the mean relative expression normalized to the untreated DL-221 control. Error bars represent the standard error of the mean (SEM) of three independent biological replicates (*n* = 3), with individual data points overlaid. Statistical significance of the treatment effect within each cell line was determined via two-way ANOVA on log_2_-transformed raw values, followed by simple main effects testing within each cell line with Bonferroni correction to account for multiple cell line testing. ***, *P* < 0.001.

We then assessed the protein levels of YAP1, TAZ, and their downstream effectors, CTGF and CYR61, following SNS-032 treatment across three sarcoma cell lines prior to complete loss of proliferation and subsequent cell death (Fig. 4; Supplementary Fig. S3). SNS-032 treatment induced a modest overall reduction in YAP1 (two-way ANOVA: treatment effect *P* = 0.032). Although baseline YAP1 expression varied (cell line effect *P* = 0.008), the interaction term was not significant (interaction *P* = 0.862). Although SNS-032 induced a 38.2% to 55.3% reduction in YAP1 across the cell lines, these effects did not reach statistical significance within any individual cell line following multiple comparisons correction. No significant overall change was detected in TAZ (treatment effect *P* = 0.055, cell line effect *P* = 0.139). Although a 40.4% to 64.2% decrease was measured in the mean TAZ expression within each cell line, the variance of replicates limited the statistical significance. In contrast, SNS-032 treatment strongly reduced the downstream transcriptional targets CTGF and CYR61. CTGF exhibited a highly significant reduction in expression (treatment effect *P* < 0.001), with baseline expression varying (cell line effect *P* = 0.010) and a nonsignificant interaction (*P* = 0.078). Across the cell lines, CTGF was reduced by 92.9% to 98.4%, which was significant within each cell line (all adjusted *P* < 0.001). Similarly, CYR61 expression was greatly reduced (treatment effect *P* < 0.001), alongside significant baseline differences (cell line effect *P* < 0.001). Although the interaction term in CYR61 was significant (*P* < 0.001), indicating differential sensitivity among cell lines, the expression decrease ranged from 95.1% to 98.8% (all adjusted *P* < 0.001), suggesting consistent near-total depletion across these cell lines.

We thus demonstrate that this large drug screen can elucidate drug classes in which sarcoma treatment investigation should continue, such as aurora kinases and CDK inhibitors. Future efforts should focus on how these sensitivities can be combined for improved translation of these findings.

## Discussion

We set out to identify drugs that could be repurposed for treating patients with sarcoma by testing a panel of sarcoma cell lines with compounds that are either FDA-approved or currently used in clinical trials. Several observations can be made from this large-scale screen. First, very few compounds were effective in these sarcoma lines, underscoring the difficulty in identifying novel antineoplastic agents from chemical screens designed primarily for carcinomas. The majority of agents shown to be effective in sarcoma cell lines have not yet been extensively tested in patients battling sarcoma. Dinaciclib, for example, has been given to two patients with sarcoma (NCT00871663), one with stable disease (25). In the gemcitabine and vinorelbine first-line therapy study (NCT00134641), five patients had an objective response (three with metastatic leiomyosarcoma, one with high-grade myxofibrosarcoma, and one with metastatic small round blue cell malignancy; ref. 26). The aurora kinase inhibitor AT9283 was used in CS (CR0708-11) with stable disease in one patient with alveolar soft-part sarcoma after four cycles (27). A broader study of the aurora kinase MLN8237 (Alisertib) in advanced and metastatic sarcoma (NCT01653028) failed to meet the primary response rate endpoint, but two partial responses were observed in angiosarcoma (28). The effectiveness of TORIN-2 in Ewing sarcoma supports previous findings (29) but has not been used in clinical trials.

Two CS, three DLPS, and the single PLS were largely resistant to most of the compounds. This resistance in CS was also observed in the recent drug screen on patient-derived tumor organoids (3). The reasons for broad resistance in sarcoma are still unclear. Recent single-cell analyses indicate that the tumor microenvironment of DLPS is highly heterogeneous, suggesting that multiple compounds may be needed to target protumor cells in the microenvironment, in addition to targeting the tumor itself (30). The nature of the extrachromosomal DNA in DLPS also enables high phenotypic plasticity that contributes to this heterogeneity (31). This genomic flexibility allows these tumors to adapt quickly to cellular assaults and external stresses imposed by these drug compounds. Therefore, carefully designed drug combinations that decrease these adaptive abilities may be required to treat sarcomas.

The CS line SW1353 was sensitive to multiple types of compounds across the drug clusters, unlike the two other CS lines, HEMC55 and CAL78. These differences were not due to mutation status in *IDH1* and *IDH2* and may represent different subgroups of CS yet to be determined, perhaps defined by overall epigenetic vulnerabilities (32, 33).

Our study was limited in its ability to extend the results to clinical variables. For instance, a screen of 194 sarcoma organoids showed that age at diagnosis and the number of prior treatments may influence response and that organoids often do not show similar efficacy to drugs with identical targets (e.g., mTOR inhibitors; ref. 3). Another strategy previously employed to identify effective treatment, termed “Probabilistic Target Inhibition Maps/PTIMs,” involves multiome profiling in genetically engineered mouse models and xenografts. This was instrumental in uncovering potential targets (34). Our study was underpowered and lacked matching omic data for such analyses.

Myxoid liposarcoma and synovial sarcoma have several shared molecular features that contribute to their shared drug response patterns. First, the IGFR/PI3K/AKT pathway is activated in these sarcomas downstream of their respective driver fusions (23, 35, 36). As the mTOR pathway is downstream of IGFR/PI3K/AKT, it follows that four of the more effective drugs for these sarcomas are mTOR inhibitors. Their driver fusions also disrupt the BAF complex. In synovial sarcoma, the fusion protein SS18::SSX displaces BAF47 in the BAF complex, resulting in a shift in the targets of the complex (37). The FUS::DDIT3 fusion in myxoid liposarcoma disrupts BAF complex binding, leading to reduced CEBP binding and, consequently, reduced expression of adipogenic genes (38). These epigenetic changes may explain the similar drug response profiles in these lines and suggest that patients bearing these sarcoma subtypes could benefit from participating in basket clinical trials targeting the BAF complex.

We observed high sensitivity to the CDK2/7/9 inhibitor SNS-032 in myxoid liposarcoma and synovial sarcoma. SNS-032 is also among the top five most effective drugs tested on tumor organoids derived from patients with sarcoma, intersecting with the top 10% most responsive organoids for each drug (3). In addition, SNS-032 has been shown to potentiate the action of mithramycin, an antineoplastic antibiotic, in Ewing sarcoma cells (39). Phase I trials of SNS-032 have already been conducted in patients with CLL and those with metastatic chemo-refractory solid tumors, with minor toxicities (40, 41). In patients with sarcoma, the CDK9 inhibitor KB-0742 is currently in trial (42). It is also well tolerated, with two patients with myxoid liposarcoma achieving clinical benefit (partial response and stable disease). Targeting CDK9 may also improve sensitivity to immune checkpoint inhibition (43). In synovial sarcoma lines treated with BS-181, a CDK7-targeting therapeutic, dose-dependent cytotoxicity was observed, along with decreases in colony formation, 3D spheroid formation, and migration (20). Increased apoptosis of these synovial sarcoma cells was also observed following CDK7 inhibition. Therefore, further investigation of CDK7 and CDK9 inhibition is warranted, especially in these two histologies.

The effectiveness of SNS-032 is partly due to its influence on YAP pathway activity, a key oncogenic pathway in these three sarcoma subtypes. Another YAP/TEAD inhibitor, verteporfin, did not achieve the same level of effectiveness in these lines in the same drug screen for several possible reasons. First, SNS-032’s activity may extend to other targets, pathways, and vulnerabilities in these subtypes. Second, our data suggest that SNS-032 reduces YAP target proteins, whereas an inhibitor may only partially block YAP-mediated oncogenic effects.

Our work is among the first to elucidate sarcoma subtypes with overlapping drug response patterns. Future investigation of these response patterns may reveal shared vulnerabilities and driver pathways that aid in repurposing drug candidates for the treatment of specific sarcoma subtypes. Further, given the widespread adoption of protein degraders in clinical development, including for the rare sarcoma subtype epithelioid hemangioendothelioma, our YAP/TAZ-related findings might point to a more effective approach that degrades YAP1, TAZ, or TEAD rather than transient protein inhibition. As many of the agents evaluated in our screen are FDA-approved or under active clinical investigation, our data shed new light on therapeutic opportunities to be exploited by pharma and academicians leading investigator-initiated studies.

## Supplementary Material

Supplemental Figure S1 Figure S1. Unsupervised hierarchical clustering of drug AUC values after filtering for min = 0.2 (blue) and max = 0.7 (red).

Supplemental Figure S2 Figure S2. Heatmap of the CDK2, CDK7, and CDK9 expression in sarcoma cells from the Cancer Cell Line Encyclopedia (CCLE) database.

Supplemental Figure S3 Figure S3. Loading control for the Western blots in Figure 3 of GAPDH for each of the myxoid liposarcoma cell lines: DDL-221, MLS-401-91, and MLS-1765-92.

Supplementary Table S1 Table S1. Filtered AUC values

Supplementary Table S2 Table S2. Top 30 ranking sensitive compound for each cell line

Supplementary Table S3 Table S3. Extraction of intersections from the upset plot (Figure 3). Cell lines are colored according to histology (color scheme from Figure 3).

## Data Availability

The data supporting these findings are available in the supplemental material and on Zenodo: https://zenodo.org/records/19098295.
