# Supplementary material for: Drug Screening of Sarcoma Cells: Finding Shared Sensitivities
Source: Cancer Res Commun. 2026 Jun 17;6(6):1425–34. doi: 10.1158/2767-9764.CRC-26-0142 (PMC13273627; doi:10.1158/2767-9764.CRC-26-0142)
Supplement: Supplemental Figure S1 — Figure S1. Unsupervised hierarchical clustering of drug AUC values after filtering for min = 0.2 (blue) and max = 0.7 (red). [file crc-26-0142_supplemental_figure_s1_suppsf1.pptx]

## Slide 1
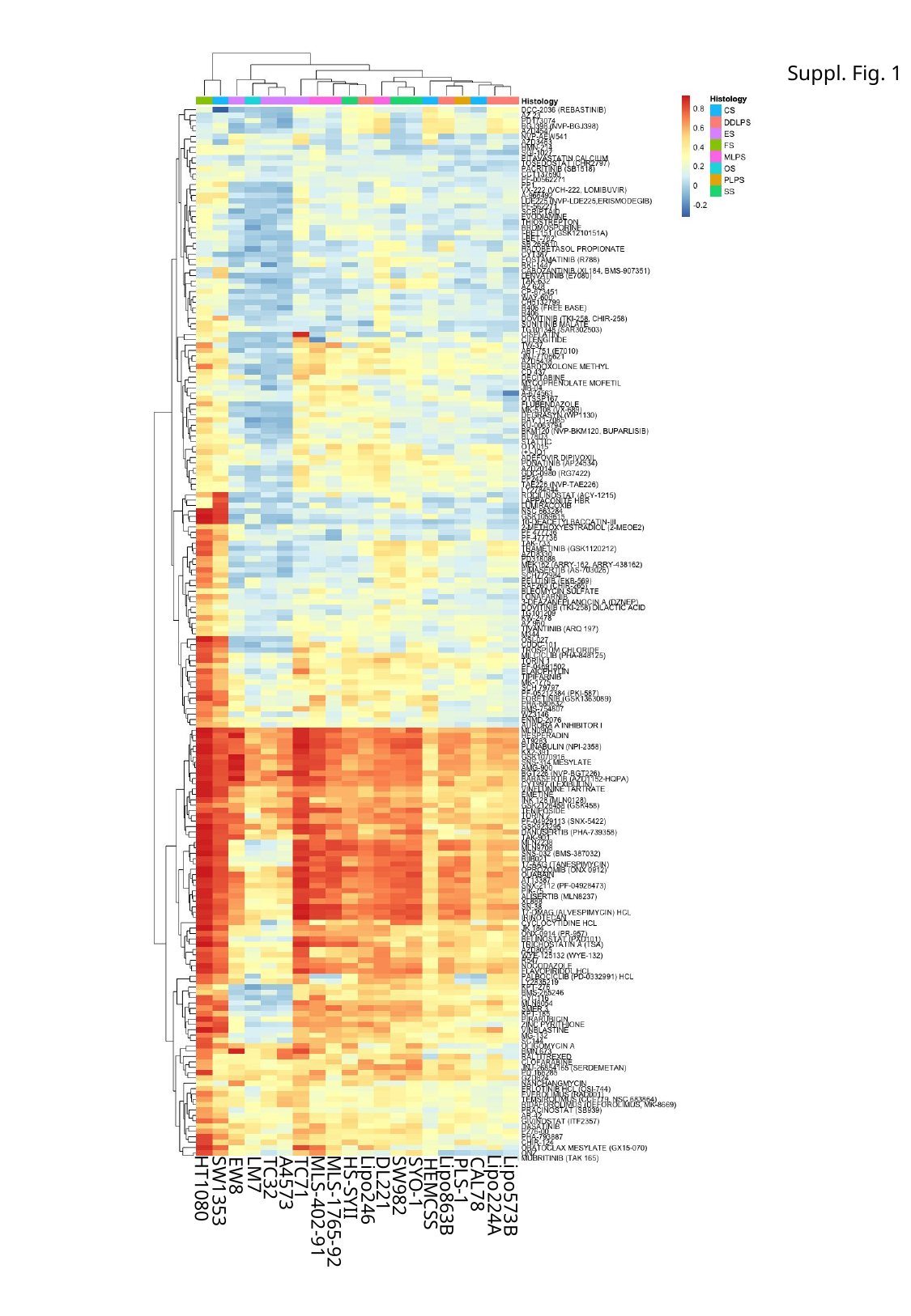

Suppl. Fig. 1
LM7
EW8
TC32
TC71
PLS-1
SYO-1
A4573
DL221
CAL78
SW982
HS-SYII
HT1080
Lipo246
SW1353
HEMCSS
Lipo224A
Lipo863B
Lipo573B
MLS-402-91
MLS-1765-92

## Slide 2
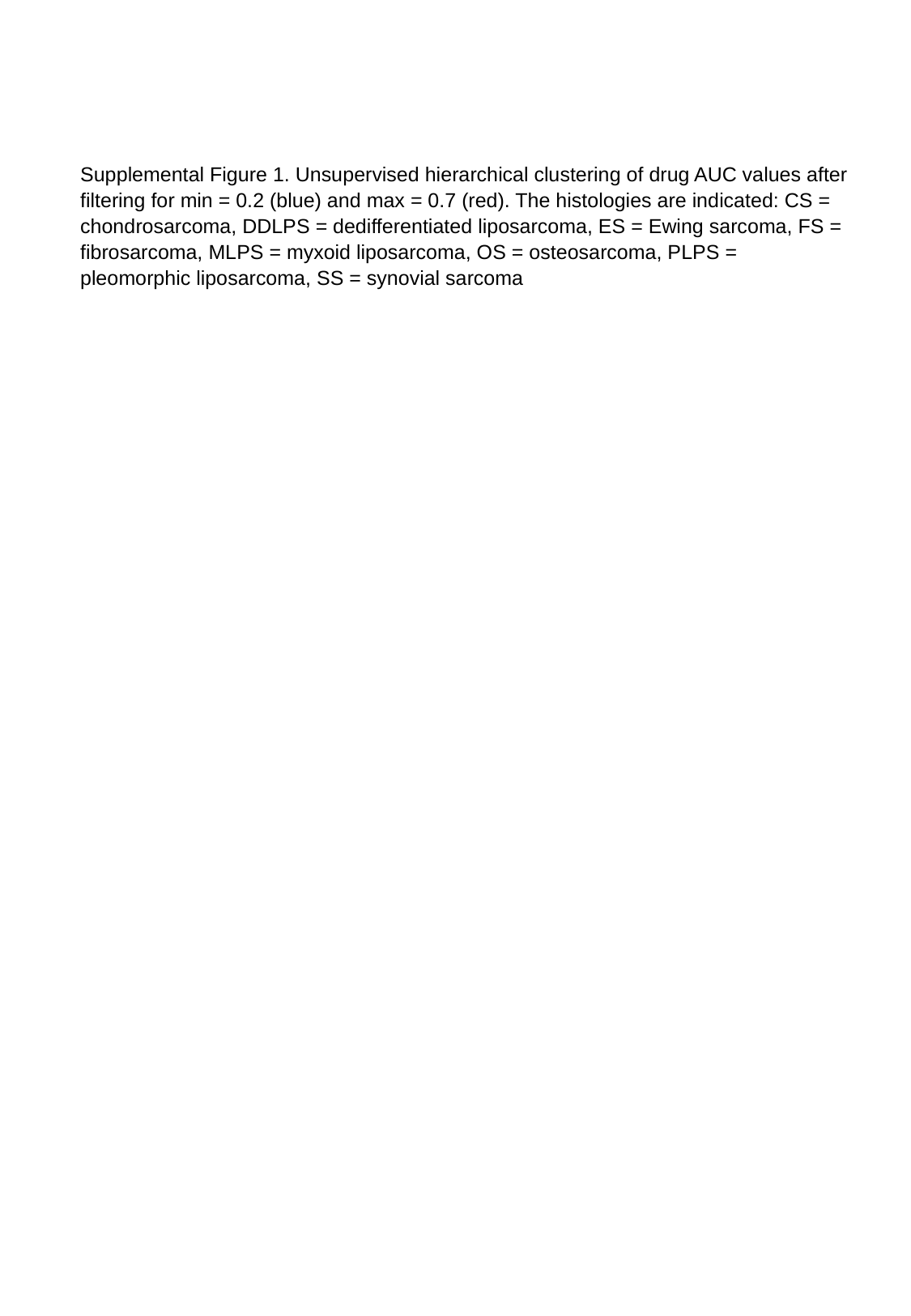

Supplemental Figure 1. Unsupervised hierarchical clustering of drug AUC values after filtering for min = 0.2 (blue) and max = 0.7 (red). The histologies are indicated: CS = chondrosarcoma, DDLPS = dedifferentiated liposarcoma, ES = Ewing sarcoma, FS = fibrosarcoma, MLPS = myxoid liposarcoma, OS = osteosarcoma, PLPS = pleomorphic liposarcoma, SS = synovial sarcoma
