# Supplementary material for: Drug Screening of Sarcoma Cells: Finding Shared Sensitivities
Source: Cancer Res Commun. 2026 Jun 17;6(6):1425–34. doi: 10.1158/2767-9764.CRC-26-0142 (PMC13273627; doi:10.1158/2767-9764.CRC-26-0142)
Supplement: Supplemental Figure S2 — Figure S2. Heatmap of the CDK2, CDK7, and CDK9 expression in sarcoma cells from the Cancer Cell Line Encyclopedia (CCLE) database. [file crc-26-0142_supplemental_figure_s2_suppsf2.pptx]

## Slide 1
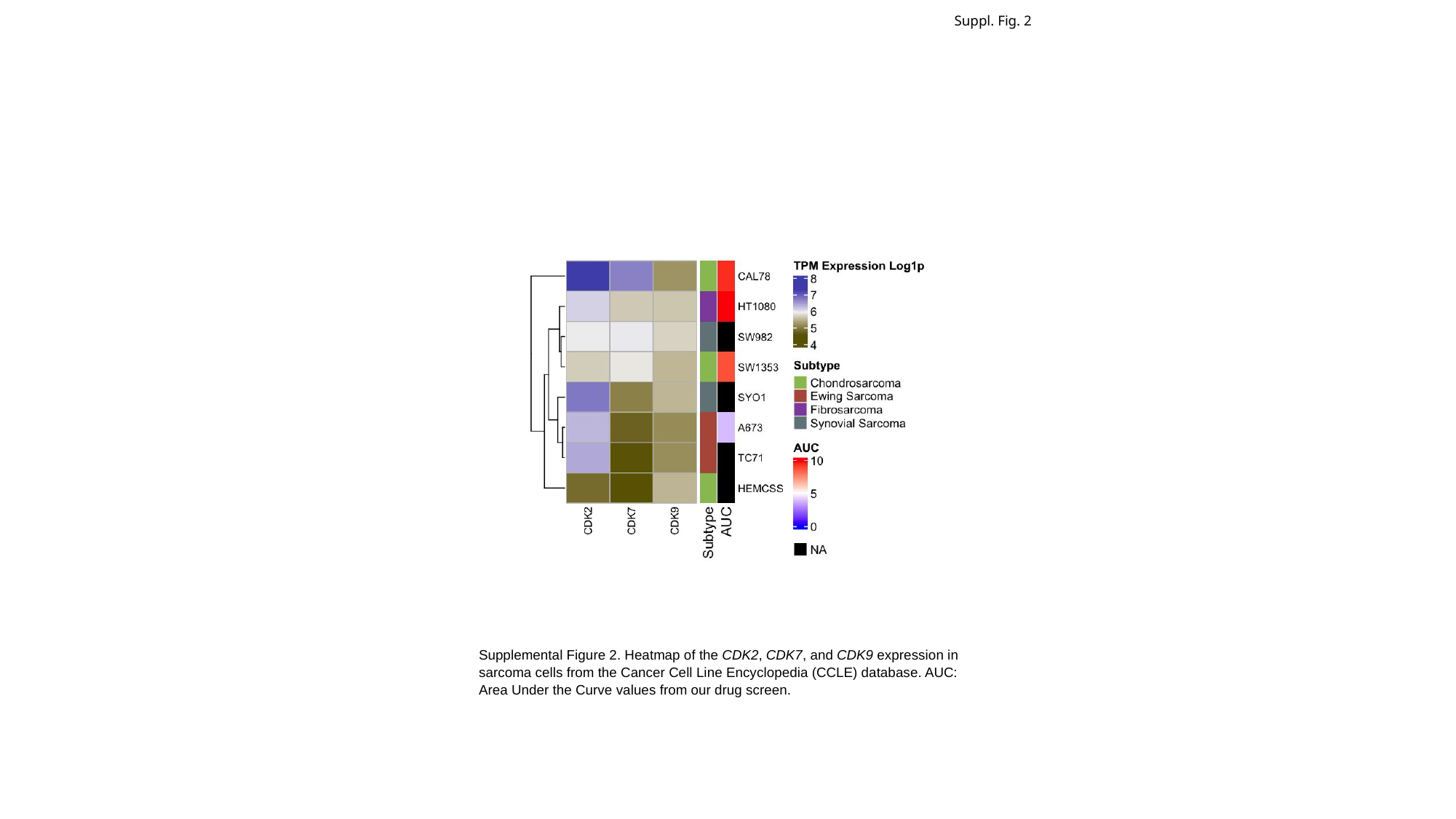

Suppl. Fig. 2
Supplemental Figure 2. Heatmap of the CDK2, CDK7, and CDK9 expression in sarcoma cells from the Cancer Cell Line Encyclopedia (CCLE) database. AUC: Area Under the Curve values from our drug screen.
