# Supplementary material for: Drug Screening of Sarcoma Cells: Finding Shared Sensitivities
Source: Cancer Res Commun. 2026 Jun 17;6(6):1425–34. doi: 10.1158/2767-9764.CRC-26-0142 (PMC13273627; doi:10.1158/2767-9764.CRC-26-0142)
Supplement: Supplemental Figure S3 — Figure S3. Loading control for the Western blots in Figure 3 of GAPDH for each of the myxoid liposarcoma cell lines: DDL-221, MLS-401-91, and MLS-1765-92. [file crc-26-0142_supplemental_figure_s3_suppsf3.pptx]

## Slide 1
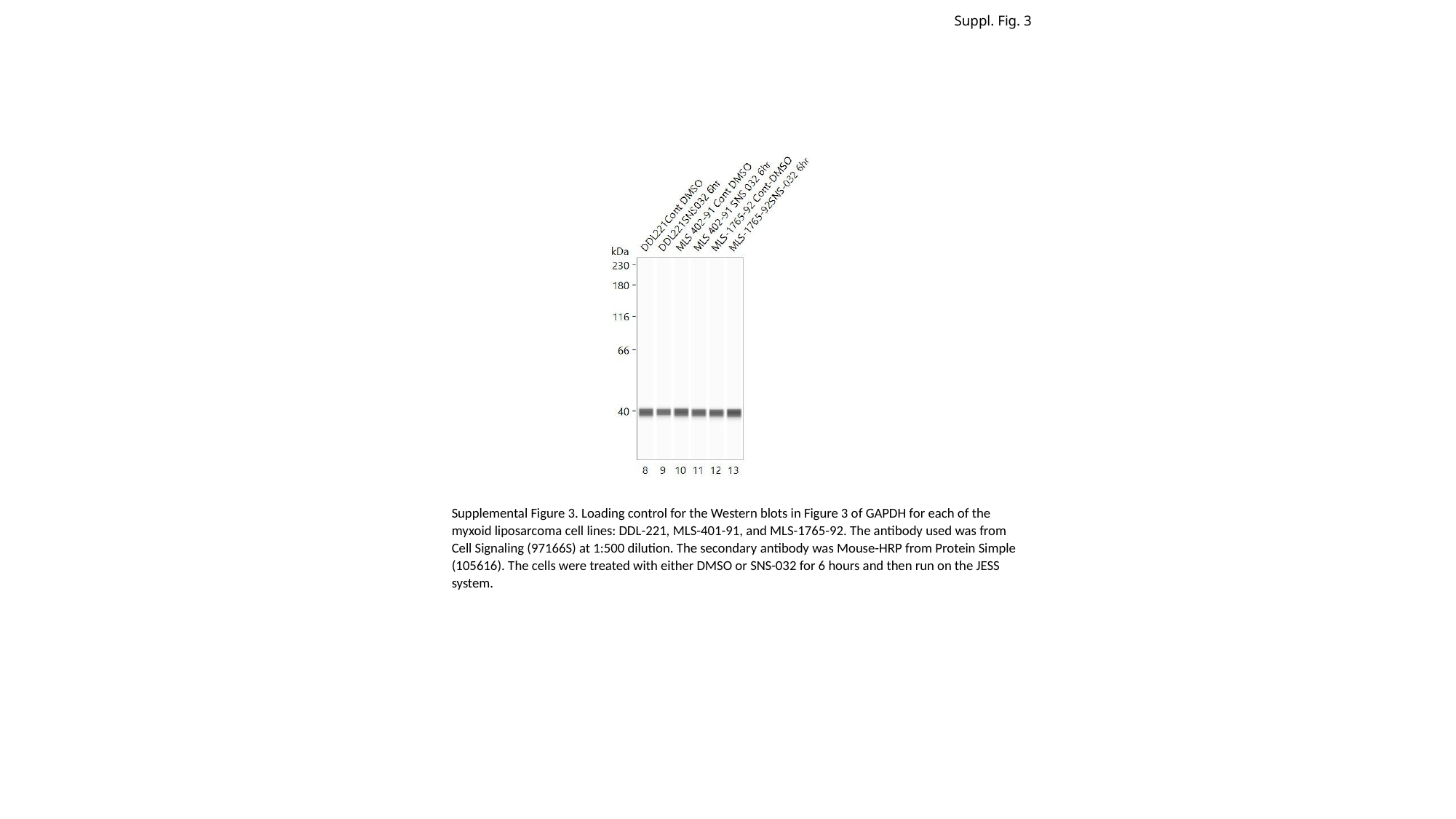

Suppl. Fig. 3
Supplemental Figure 3. Loading control for the Western blots in Figure 3 of GAPDH for each of the myxoid liposarcoma cell lines: DDL-221, MLS-401-91, and MLS-1765-92. The antibody used was from Cell Signaling (97166S) at 1:500 dilution. The secondary antibody was Mouse-HRP from Protein Simple (105616). The cells were treated with either DMSO or SNS-032 for 6 hours and then run on the JESS system.
